# Impact of methodology on estrogens’ effects on cerebral ischemia in rats: an updated meta-analysis

**DOI:** 10.1186/1471-2202-15-22

**Published:** 2014-02-04

**Authors:** Jakob O Ström, Edvin Ingberg

**Affiliations:** 1 Vårdvetenskapligt Forskningscentrum/Centre for Health Sciences, Örebro University Hospital, County Council of Örebro, Örebro SE-703 62, Sweden; 2 Clinical Chemistry, Department of Clinical and Experimental Medicine, Faculty of Health Sciences, Linköping University, Linköping, Sweden; 3 School of Health and Medical Sciences, Örebro University, Örebro, Sweden

**Keywords:** Cerebral ischemia, Estradiol, Estrogens, Meta-analysis, Rats, Stroke

## Abstract

**Background:**

Although most animal stroke studies have demonstrated potent neuroprotective effects of estrogens, there are a number of articles reporting the opposite. In 2009, we made the case that this dichotomy was related to administered estrogen dose. Several other suggestions for the discordant results have also been propagated, including the age of the experimental animals and the length of hypoestrogenicity prior to estrogen administration. These two suggestions have gained much popularity, probably because of their kinship with the window of opportunity hypothesis, which is commonly used to explain the analogous dichotomy among human studies. We were therefore encouraged to perform an updated meta-analysis, and to improve it by including all relevant variables in a large multiple regression model, where the impact of confounders could be controlled for.

**Results:**

The multiple regression model revealed an indisputable impact of estrogen administration mode on the effects of estrogens in ischemic stroke. Subcutaneous slow-release pellets differed from the injection and silastic capsule treatments in terms of impact of estrogens on ischemic stroke, showing that the first mentioned were more prone to render estrogens damaging. Neither the use of elderly animals nor the adoption of longer wash-out periods influenced estrogens’ effects on experimental ischemic stroke in rats.

**Conclusions:**

We conclude that the discordant results regarding estrogens’ effects in rat models of ischemic stroke are a consequence of differences in estrogen administration modes. These results are not only of importance for the ongoing debate regarding menopausal hormone therapy, but also have an important bearing on experimental stroke methodology and the apparent translational roadblock for suggested stroke interventions.

## Background

Estrogens’ effects in focal cerebral ischemia have been a matter of debate for more than a decade. Among studies performed in humans, the early epidemiological studies, indicating decreased stroke incidence from peri-menopausal hormone therapy [1-3], starkly contrasted later randomized controlled trials, such as the Women’s Health Initiative [4]. In an effort to explain the discrepant results, the window of opportunity hypothesis has been propagated, stating that estrogen therapy may be detrimental after a longer period of hypoestrogenicity [5]. To complement the human studies, a large number of animal studies have been devoted to investigate the impact of estrogens on artificially induced ischemic stroke. Even if the vast majority of animal studies have reported protection from estrogens [6,7], there are some studies where the hormone has been observed to increase infarction [8,9], thus paralleling the dichotomy among human studies. The most popular suggestions to explain the discordant results in animal studies has, in analogy with the human studies, been that differences in length of wash-out time between ovariectomy and estrogen administration or animal age are the responsible factors [10,11].

In 2009, a systematic analysis from our lab suggested that the use of different estrogen administration modes may explain the dichotomous results from the animal studies [12]. Although such a hypothesis may seem less attractive because it is not readily extrapolated to the human studies, where the range of tested dosages and administration modes is extremely narrow, the fact that all animal studies that reported damage from estrogen used the same type of estrogen administration seemed an unlikely coincidence. In the studies included in the former systematic analysis, the only administration mode capable of rendering estrogens damaging was a type of subcutaneous slow-release pellet (produced by the company Innovative Research of America^®^), which has been demonstrated to produce extremely high, prolonged serum 17β-estradiol concentrations in rodents [13-15]. However, in the 2009 systematic analysis, separate analyses handled one variable at a time, and thus confounders were not controlled for. The discussion regarding what factors contribute to the estrogen-stroke dichotomy has nevertheless proceeded, and for example in a recent review, Sohrabji et al. suggested that the factors age, hypertension, rat strain and whether the middle cerebral artery occlusion (MCAo) was permanent or not could explain why some articles report enlarged infarcts from estrogen administration. The fact that slow-release pellets had been used in all cited studies in which estrogens had increased damage was not even mentioned [16].

The statistical shortcomings of the previous systematic analysis, the continuing debate on the matter and the publication of several additional original studies since the previous systematic analysis encouraged us to perform an updated and improved meta-analysis, where all methodological differences that reasonably could affect the impact of estrogens on experimental ischemic stroke would be controlled for in a large multiple regression model. The current meta-analysis therefore aimed to address the hypotheses that (A) estrogen administration mode, (B) the age of the experimental animals and (C) the length of hypoestrogenicity affects estrogens’ impact on stroke.

## Results

### Slow release pellets render estrogens significantly less protective/more damaging

**Figure 1:**
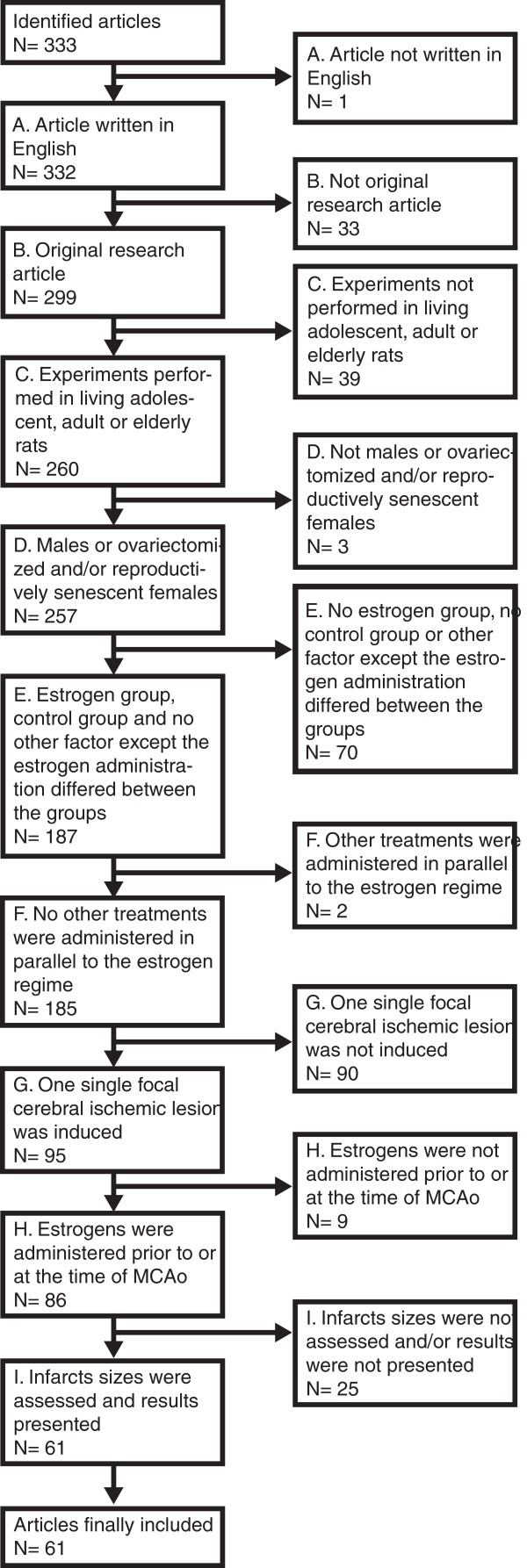
**Three hundred and thirty-three articles were assessed for inclusion according to criteria A to I.** Consensus was reached to finally include 61 articles, of which 45 were included in the previous systematic analysis, and 16 were not [12].

**Figure 2:**
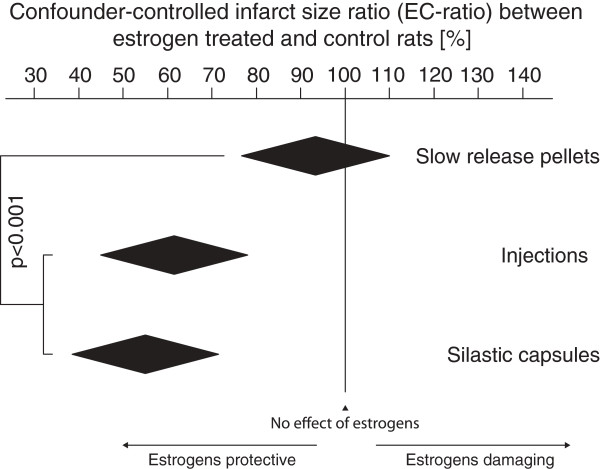
**After controlling for confounding factors, it remained clear that slow-release pellets rendered significantly higher EC-ratios than the injection and silastic capsule regimens did (p < 0.001).** The black diamonds mark the mean EC-ratio with 95% confidence interval for each of the three administration modes. Of note, the slow-release pellet diamond reaches over the 100% EC-ratio line, demonstrating the pellets’ potential for harm as well as protection. EC-ratio = infarct size in estrogen treated group divided by infarct size in control group.

Sixty-one studies, describing 124 pairs of estrogen-treated groups and control groups (subsequently referred to as “group pairs”) of rats in which focal cerebral ischemia was induced, were included (Figure 1), and data regarding methodology and results was extracted for meta-analysis (Table 1). In the final multiple regression model, with correction for all included confounding factors, the effects of estrogens on focal cerebral ischemia was clearly affected by the mode of estrogen administration. Slow-release pellets rendered estrogens significantly more damaging/less protective than if the estrogens were administered via injections or silastic capsules (p < 0.001; Figure 2, Table 2). The use of direct, mechanical MCAo procedures, laser-Doppler flowmetry surveillance, edema correction and the variable *Length of time between initiation of estrogen administration and induction of the ischemic damage* were also found to significantly influence the impact of estrogens on focal cerebral ischemia. Notably, concerning the abovementioned hypotheses B and C, the factors *Elderly rats* and *Washout* were excluded in the preceding Backward analysis due to too low impact on the outcome variable *Infarct size ratio between estrogen treated and control rats* (subsequently referred to as “EC-ratio”; Table 2).

**Table 1:** Extracted variables

**Factor/outcome measure**	**Data type**	**Final categories/unit**	**Reference category for regression analyses**
**Rat properties**			
*Strain*	Category	I. *Sprague Dawley*	*Sprague Dawley*
		II. *Wistar*	
		III. *Other strains*	
*Sex*	Category	I. *Female*	*Female*
		II. *Male*	
*Diseases*	Category	I. *Healthy*	*Healthy*
		II. *Diabetes*	
		III. *Hypertension*	
*Elderly rats*	Category	[No]	[No]
		[Yes]	
*Number of rats in estrogen treated and control groups*	Continuous		NA
**Estrogen administration**			
*Estrogen administration mode*	Category	I. *Slow-release pellets*	*Slow-release pellets*
		II. *Injections*	
		III. *Silastic capsules*	
*Estrogen type*	Category	I. *17β-estradiol*	*17β-estradiol*
		II. *Estradiol valerate*	
		III. *Premarin*	
		IV. *Estrone*	
*Slow-release pellets: estrogen dose/pellet*	Continuous	μg	NA
*Injections: Daily estrogen dose*	Continuous	μg/kg body weight	NA
*Silastic capsules: estrogen dose/silastic capsule*	Continuous	μg	NA
*Washout (Length of time between ovariectomy and estrogen administration)*	Category	I. *0*–*14 days*	*0-14 days*
		II. *>14 days*	
*Length of time between initiation of estrogen administration and induction of the ischemic damage*	Continuous	Hours	NA
**Focal ischemia procedure**			
*Type of middle cerebral artery occlusion procedure*	Category	I. *Intraluminal filament*	*Intraluminal filament*
		II. *Direct, mechanical*	
		III. *Embolic*	
		IV. *Photothrombotic*	
		V. *Endothelin injection*	
*Occlusion duration*	Category	I. *Permanent*	*Permanent*
		II. *Short transient (up to 60 minutes)*	
		III. *Long transient (>60 min)*	
*Laser-Doppler flowmetry during surgery*	Category	[No]	[No]
		[Yes]	
**Analysis procedure**			
*Length of time between ischemia and evaluation of damage Edema correction*	Continuous	Hours	NA
*Edema correction*	Category	I. *No edema correction used*	*No edema correction used*
		II. *Correcting for edema in infarct*	
		III. *Correcting for edema in entire hemisphere*	
**Outcome measures**			
*Infarct size ratio between estrogen treated and control rats* – “EC-ratio”	Continuous	%	NA

**Table 2:** Multiple regression analysis

**Variable (reference category)**	**Variable categories**	**Regression coefficient**	**0.95 Confidence interval for regression coefficient**		**p-value**
			**Lower bound**	**Upper bound**	
Constant/baseline – showing the effect of the reference categories	NA	93.4	76.7	110.1	
*Estrogen administration mode (Slow-release pellets)*	*Injections*	-31.9	-48.5	-15.2	0.000
	*Silastic capsules*	-38.2	-54.9	-21.6	0.000
	*Per oral*	-59.3	-129.3	10.7	0.096
*Type of middle cerebral artery occlusion procedure (Intraluminal filament)*	*Direct, mechanical*	33.2	18.8	47.6	0.000
	*Embolic*	-42.0	-90.8	6.7	0.091
	*Photothrombotic*	20.6	-53.1	94.2	0.581
	*Endothelin injection*	2.3	-31.2	35.9	0.891
*Laser-doppler flowmetry during surgery (No)*	[Yes]	-15.3	-29.4	-1.2	0.034
*Edema correction (No edema correction used)*	*Correcting for edema in infarct*	-21.8	-54.2	10.6	0.184
	*Correcting for edema in entire hemisphere*	-23.1	-39.6	-6.7	0.006
*Length of time between initiation of estrogen administration and induction of the ischemic damage*	Continuous; hours	0.025	0.011	0.040	0.001

Variables excluded in preceding multiple regression analysis (with backward exclusion) due to too low explanatory value: *Strain, Sex, Elderly rats, Washout (Length of time between ovariectomy and estrogen administration), Occlusion duration, Length of time between ischemia and evaluation of damage.*

The final multiple regression model included 124 group pairs, and yielded an r^2^-value of 0.484, hence explaining 48.4% of the variation in EC-ratio.

### Higher dose in slow-release pellets increases the risk of estrogen exacerbating ischemic damage

**Figure 3:**
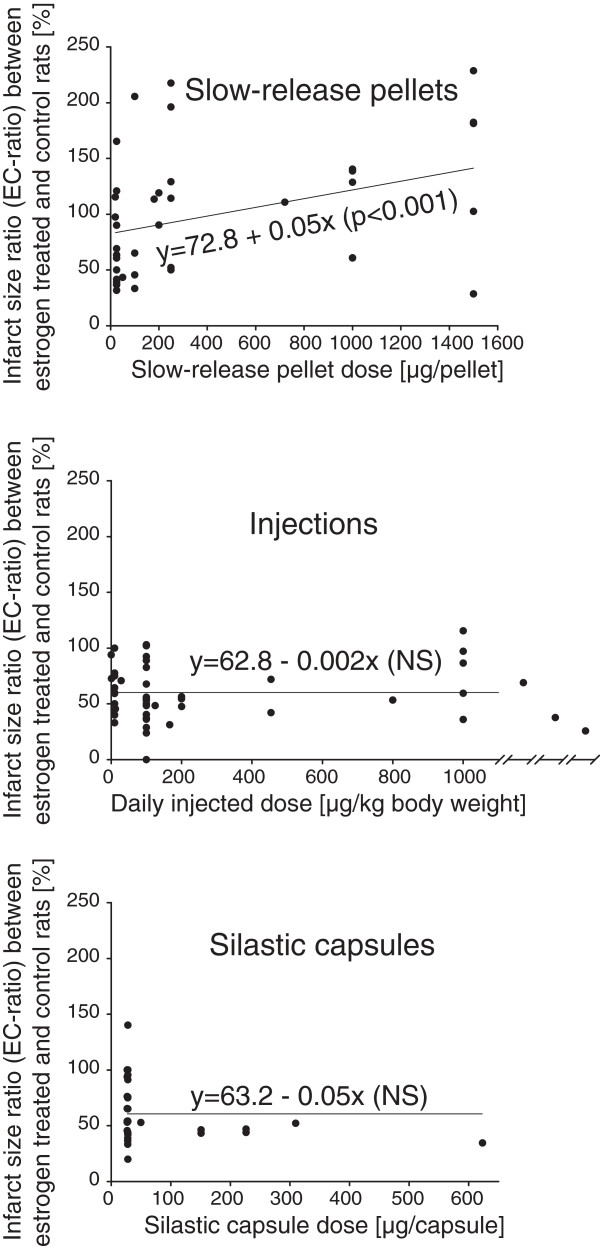
**By three simple linear regression analyses, it was found that higher pellet doses significantly increased EC-ratio (y = 72.8 + 0.05x; p = 0.001), while there was an opposite trend (not significant) among injection and silastic capsule regimens.** The numbers of group pairs included in the slow-release pellet, injection and silastic capsule simple regression models were 39, 50 and 31, respectively. Note that the injection graph has been compressed in the upper dose range to accommodate three group pairs being administered very high doses.

Simple linear regression analyses including only group pairs within one specific estrogen administration mode category were run with dose (μg/pellet, daily injected dosage in μg and μg/silastic capsules, respectively) as the independent factor and EC-ratio as the outcome variable. There was a significant, positive relation between slow-release pellet dose and EC-ratio (y = 72.8 + 0.05x; p = 0.001; N = 39), meaning that increasing the pellet dose increased the likeliness that estrogens would be damaging. This model had an r^2^-value of 0.26, indicating that it explained 26% of the EC-ratio variation (Figure 3). Adding the factor *Elderly rats* (to address hypothesis B) to the model did not affect the r^2^-value (0.26), and there was not even a slight tendency for increased damage in elderly rats (regression coefficient -2.9, with confidence intervals -56.1 to 50.2). A similar analysis to address hypothesis C could not be done since only one single study administered estrogen via pellets more than 14 days after ovariectomy.

For the group pairs administered estrogens via injection or silastic capsules, there were no significant relations between dosage and EC-ratio, however the trend in both models was that increased estrogen dose increased neuroprotection (Injection dose vs. EC-ratio: y = 62.8-0.002x; p = 0.14; N = 50; Silastic capsule dose vs. EC-ratio: 63.2-0.05x; p = 0.17; N = 31).

### Descriptive statistics

**Figure 4:**
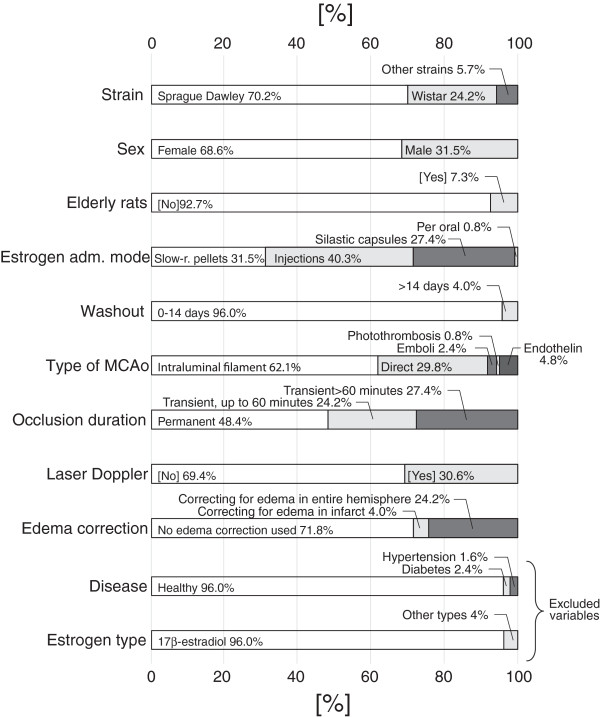
**The frequencies of the different categories in the 124 group pairs are presented as percentages.** Please note that the Disease and Estrogen type variables were omitted from the statistical analysis because too few studies used diseased animals and other estrogens than 17β-estradiol, respectively.

The frequencies of categories in the 124 included group pairs are depicted in Figure 4. Two variables, *Diseases* and *Estrogen type*, were excluded from the statistical analyses because too few group pairs deviated from the dominant category. Descriptive statistics for the continuous variables are presented in Table 3.

**Table 3:** Central tendencies and dispersion measures of registered continuous variables

**Variable**	**Unit**	**Mean**	**Standard deviation**	**Median**	**Inter-quartile range**
*Total number of rats in estrogen treated and control groups*		16.0	7.3	15	10-20
*Slow-release pellets: estrogen dose/pellet*	μg	412.6	53.0	180.0	25.0-1000.0
*Injections: daily estrogen dose*	μg/kg body weight	794.8	3006.2	100.0	11.9-200.0
*Silastic capsules: estrogen dose/silastic capsule*	μg	77.5	124.8	28.0	27.2-28.0
*Length of time between initiation of estrogen administration and induction of the ischemic damage*	Hours	221.6	600.5	168	0.5-324
*Length of time between ischemia and evaluation of damage*	Hours	49.6	96.4	24.0	24.0-24.0
*Infarct size ratio between estrogen treated and control rats*	%	72.1	42.3	56.2	43.7-93.9

## Discussion

The main multiple regression model, controlling for all listed confounders, revealed an indisputable impact of estrogen administration mode on the effects of estrogens in ischemic stroke (p < 0.001). Slow-release pellets significantly differed from the injection and silastic capsule treatments in terms of resulting EC-ratio, showing that slow-release pellets are more prone to render estrogens damaging. Of note, the slow-release pellet confidence interval extended over the 100% EC-ratio line, underscoring the potential for harm as well as benefit (Figure 2, Table 2). In an attempt to further shed light on this finding, administration mode-specific simple regression models between dose and EC-ratio were run. While no statistically significant relation was seen between dose and EC-ratio in the injection and silastic capsule group pairs, higher slow-release pellet dose was clearly related to increased ischemic damage (Figure 3).

A plausible explanation for the tendency of estrogens in slow-release pellets to be less protective/more damaging is that this high-dose administration mode causes extremely high, elevated serum 17β-estradiol concentrations. It has repeatedly been shown that the pellets from Innovative Research of America^®^, albeit with a large portion of unpredictability, render prolonged serum concentrations that are well beyond the physiological spectrum. Silastic capsules produce serum concentrations in a much lower, often physiological, range, while daily injections result in hours-long spikes followed by estrogen-deficient intervals, rendering the 24 h-average serum concentrations low [13-15]. The finding that higher pellet dose increased the detrimental tendency (Figure 3) is well in line with the hypothesis that the administered dose is the culprit. Such an explanation is reflected in the concept of *hormesis,* stating that dose–response curves are not always unidirectional, but that a substance can have diametrically different effects in different concentration ranges [17,18]. The current meta-analysis strongly suggests that low-dose estrogen therapy is protective in rat models of focal cerebral ischemia, while high-dose estrogen therapy is more likely to be detrimental. Recently, a study aiming to experimentally test this hypothesis was published in BMC Neuroscience. The main finding was that true hormesis, with bidirectional effects in different dose ranges compared to baseline, could be demonstrated in the in vitro oxygen-glucose deprivation experiments. When different doses of 17β-estradiol in analogy were tested on rats, higher doses rendered less protection than low doses [19]. While there is a multitude of narrative reviews covering the field of animal models of stroke and estrogens, to the best of our knowledge only one systematic review, except for the 2009 article from our lab referred to in the Background, has been published. The main finding presented in that article was that estrogens seemed to reduce ischemic damage in a positive dose-dependent manner [20], a result that could not be corroborated by the current review. The previous review differed fundamentally from the current meta-analysis by not analyzing differences between studies reporting neuroprotection versus studies reporting increased damage from estrogens.

Regarding hypothesis B, no impact of the use of elderly animals on estrogens’ effects in stroke was seen. In the preceding Backward multiple regression analysis, the variable was the first one to be excluded, without even a trace of higher EC-ratio in elderly rats (regression coefficient before exclusion: -2.6, 95% confidence intervals -29.2 to 24.0; p = 0.85). If anything, the regression coefficient suggested *more* estrogenic protection in elderly animals, in contrary to hypothesis B.

Similarly for hypothesis C, the second (dummy) variable to be excluded in the Backward analysis was the wash-out category *>14 days* (regression coefficient before exclusion: -6.6, 95% confidence intervals -44.1 to 30.9; p = 0.73). In analogy with the use of elderly rats, the regression coefficient pointed in the negative direction, negating hypothesis C by indicating *more* protection from estrogen after longer hypoestrogenic intervals. It however deserves mention that since elderly rats or wash-out periods beyond 2 weeks were used in a low number of studies, conclusions about these variables should be drawn with caution (Figure 4). Although no support for hypotheses B and C was found in the present analysis, researchers have previously suggested mechanisms for the detrimental effects of high age and long periods of hypoestrogenicity. For example in an article published 2010, Selvamani et al. put forward that decreasing IGF-1 levels in elderly rats and further suppression by estrogen treatment is associated with a decline in estrogens’ beneficial effects in the aging brain [11]. Regarding the potential harm of a prolonged wash-out period, Suzuki et al. proposed 2007 loss of anti-inflammatory actions of estrogens as a possible mechanism [10].

A few other variables were found to significantly affect the EC-ratio, however since these did not address the main hypotheses, we refrain from drawing conclusions about them, and refer to Table 2 for the interested reader.

### Strengths and weaknesses

An inherent draw-back with linear regression analysis is that linear relations are assumed, which evidently is not always true. This imperfection must be kept in mind when assessing the results. Another weakness of the current study is that the 124 group pairs were described in only 61 articles, which in turn were published by even fewer research groups. To be perfectly stringent, group pairs from the same article or the same research group should not be regarded as independent. However, creating dummy variables for each article or research group would have made the analysis totally devoid of statistical power, and thus impossible to perform.

The analysis presents a composite result of data gathered from 124 group pairs and more than 1900 rats, handled in a rich variety of experimental conditions. The main strength of the current meta-analysis is that all factors that have been suggested to be responsible for the discrepant results were tested in parallel, thus potently correcting for confounders.

## Conclusions

We conclude that the discordant results regarding estrogens’ effects in rat models of ischemic stroke are a consequence of differences in estrogen administration modes, corroborating the earlier systematic analysis from our laboratory [12]. The clearly protective effects of silastic capsules and injections contrast the potentially harmful effects of estrogens delivered via the high-dose slow-release pellets. Further, the relation between higher pellet doses and increased propensity for causing damage underscores the plausibility that delivered dose is the culprit, which is reflected in the concept of hormesis. The meta-analysis could not corroborate the hypotheses that (B) the age of the experimental animals nor (C) the length of hypoestrogenicity affected estrogens’ impact on stroke.

## Methods

### Article search and inclusion

To define articles to include in the meta-analysis, Medline was the 5^th^ of March 2013 searched with the search line *(stroke OR “cerebral ischemia” or “brain infarction” or “cerebral infarction” or “brain ischemia” or mcao or “middle cerebral artery occlusion”) AND (estrogen OR estradiol OR estrogens) and rat*. The articles retrieved by the search together with the articles included in the previously mentioned systematic analysis, in total 333 papers, were independently assessed for inclusion by two researchers. A specific study was included if all the following criteria were met:

A. Article written in English

B. Original research article

C. The experiment was performed in adolescent, adult or elderly rats

D. The rats were males or ovariectomized and/or reproductively senescent females

E. Each estrogen treatment group had a corresponding control group, and no factor except the estrogen administration per se differed between the groups

F. No other treatments were administered in parallel to the estrogen regime

G. One single focal cerebral ischemic lesion was induced in the animals

H. The estrogens were administered prior to or at the time of MCAo

I. Infarct sizes were assessed and results presented

Consensus was reached to include 61 studies, of which 45 were included in the previous systematic analysis [6,8,9,21-62] while 16 were not [11,63-77]. The previous systematic analysis [12] included not only focal cerebral ischemia, but also global ischemia, which is really not a model of ischemic stroke, but rather models the effects of cardiac arrest on the brain. Also, only studies using adult rats were included in the current study, since there are relevant differences in brain pathophysiology as well as in models for cerebral ischemia between adult animals and pups. These changes to make the inclusion criteria more scientifically stringent explain why only 45 of 66 articles from the previous study were used in the current study. Several articles included more than one pair of an estrogen-treated group and a corresponding control group, differing in for example euthanasia time-point. All these group pairs were separately included and assessed independently of each other. In the 61 studies, 124 pairs of estrogen-treated groups and control groups were identified.

It was initially the intention to expand the meta-analysis by setting up parallel models for studies in mice. However, the search line *(stroke OR “cerebral ischemia” or “brain infarction” or “cerebral infarction” or “brain ischemia” or mcao or “middle cerebral artery occlusion”) AND (estrogen OR estradiol OR estrogens) and (mice or mouse)* only identified 22 articles eligible for inclusion, with too few group pairs to substantiate the planned multiple regression analysis. Further, in only one single group pair, after isoflurane-preconditioning, estrogens were found to increase damage from focal cerebral ischemia. Therefore, it was decided to let rat studies be the sole focus of this article.

### Data extraction

Eighteen pre-defined method and result variables were extracted from the 61 articles describing the 124 group pairs. Method variables were chosen with the aim to encompass all methodological aspects that theoretically could influence estrogens’ effects on ischemia. When extracting the method data, the principle “If it was not described, it was not performed” was strictly adhered to. All extracted variables are presented in Table 1. When possible, categories represented by less than 5 group pairs were in the analysis clumped up in an *Others* category. Further, some other reductions in number of categories were performed, as presented under “Variable definitions and cathegorizations” below.

### Processing of data

#### Variable definitions and categorizations

Sprague Dawley and Wistar were the only strains that were sufficiently well-used (at least 5 group pairs) to deserve separate categories. All other registered strains (Lister hooded rats, Diabetes type 1 rats, Spontaneously hypertensive rats – stroke prone, Spontaneously hypertensive rats and rats of unknown strain) were put in an *Other strains* category.

The category *Males* in the *Sex* variable was let to include both castrated and intact male animals, since castrated males were used in too few studies (n = 3) to deserve a separate category.

Too few studies used diseased animals for the *Disease* variable to be meaningfully included in the meta-analysis. The variable was therefore omitted from the statistical analysis, but is for the sake of transparency presented in Figure 4.

*Elderly rats* were defined as being at least 9 months of age at time of ischemic insult.

The continuous variable *Number of rats in estrogen treated and control groups* was calculated by simply summarizing the number of animals included in the infarct size measurements in each group pair.

Slow-release pellets from the company Innovative Research of America^®^, various injections and subcutaneous silastic capsules defined the three *Estrogen administration mode* categories. The *Injection* category consisted mainly of subcutaneous treatments, but a few group pairs included in this category were treated with intramuscular, intravenous and intraperitoneal injections. Since the other routes than subcutaneous were each too small to define separate categories, they were all included in the injection category. It can be argued that the pharmacokinetic differences between the injection regimens are too large to justify grouping them; however, they do share the important common characteristic of a high plasma concentration peak with short duration, which was why it was still deemed a relevant category [13]. Only one group pair was treated with 17β-estradiol orally, which was far too few for meaningful analysis. However, since no other “superfluous” categories in this variable existed, the single study was put in a separate Oral treatment category.

The variable *Estrogen type* was omitted since only five groups were treated with other estrogen forms than 17β-estradiol. Premarin was used in two goups, estradiol valerate in one group and estrone in two groups. The variable is nevertheless presented in Figure 4.

The three variables *Slow-release pellets: estrogen dose/pellet*, *Injections: Daily estrogen dose* and *Silastic capsules: estrogen dose/silastic capsule* presented in Table 1 were not included in the main multiple regression model, since each of these variables was only relevant for a limited number of group pairs. These categories were instead used in subsequent simple linear regression models.

To deal with the fact that male rats are not ovariectomized, and that no fields in the multiple regression analysis are allowed to be empty, the variable *Washout (Length of time between ovariectomy and estrogen administration)* was first separated into two categories, *0*–*14 days* and *>14 days*, and then combined with the variable *Sex*, making *Males* a third category.

In the variable *Type of middle cerebral artery occlusion procedure*, the category *Direct, mechanical* refers to all MCAo procedures where the MCA was mechanically occluded from the outside, for example by clips, cauterization or ligation. The categories *Emboli* and *Photothrombosis* included only 3 and 1 group pairs, but were treated according to the same principles as the *Oral treatment* category above.

Regarding the *Occlusion duration* variable, only methods including actions taken to ensure reperfusion (such as removing the occluding intraluminal filament or arterial clip) were considered transient.

Regarding the category *Laser-Doppler flowmetry during surgery*, group pairs were put in the [Yes]-category irrespective of whether or not it was explicitly described that the laser-Doppler was used to exclude animals.

Edema correction when calculating infarcts sizes can be performed according to at least two principles. Swanson et al. [78] described a procedure that focuses on the loss of viable tissue instead of on the infarct area per se:

$$\left[\mathrm{Corrected}\;\mathrm{infraction},\mathrm{as}\;\mathrm{part}\;\mathrm{of}\;\mathrm{one}\;\mathrm{hemisphere}\right]=\frac{\left[\mathrm{Total}\;\mathrm{contralateral}\;\mathrm{hemisphere}\right]-\left[\mathrm{Viable}\;\mathrm{tissue}\;\mathrm{in}\;\mathrm{infarcted}\;\mathrm{hemisphere}\right]}{\left[\mathrm{Total}\;\mathrm{contralateral}\;\mathrm{hemisphere}\right]}$$

This method assumes that all edema is in the infarct, and not outside it, and defined the category *Correcting for edema in infarct.* An alternative is to express the infarction as a percentage of the ipsilateral hemisphere:

$$\begin{array}{l} \left[\mathrm{Corrected}\;\mathrm{infarction},\mathrm{as}\;\mathrm{part}\;\mathrm{of}\;\mathrm{one}\;\mathrm{hemisphere}\right]\\ \;=\frac{\left[\mathrm{Crude}\;\mathrm{infarct}\;\mathrm{area}\right]}{\left[\mathrm{Total}\;\mathrm{ipsilateral}\;\mathrm{hemisphere}\right]} \end{array}$$

The assumption here is instead that the edema is equally distributed in the entire infarcted hemisphere, and group pairs in which this procedure had been adopted were registered in the category *Correcting for edema in entire hemisphere*.

The outcome variable *Infarct size ratio between estrogen treated and control rats* (in the text referred to as “EC-ratio”) was calculated by simply dividing the mean infarct volume in the estrogen treated group by the mean infarct volume in the control group, and then multiplying by 100 to yield a percentage. Hence, a percentage above 100% means that the estrogen group on average suffered from larger (nominally; whether significant or not) infarctions than the control group, while numbers below 100% indicates the opposite. For example, a group pair in which estrogen treatment halved the infarct size would obtain an EC-ratio of 50%.

#### Statistical analyses

To identify which methodological factors significantly affected the EC-ratio, multiple linear regression analysis was used. As abovementioned, this analysis was the most important improvement from our previous systematic analysis [12]. While the different methodological variables were analyzed separately in the previous article, the current meta-analysis combined them all in a large multiple regression model. In this main model, EC-ratio was the outcome variable, *Number of rats in estrogen treated and control groups* attributed each group pair a weight in the analysis, and all other extracted variables (except the aforementioned excluded ones, and the administration mode-specific dose variables) were considered independent factors. All category variables were dummy-converted before analysis. The main multiple regression analysis consisted of two steps. First, a multiple regression model with Backward exclusion of variables (p-value set to 0.10 for exclusion) was performed to eliminate independent factors that did not significantly contribute to the model (the variables excluded due to too low explanatory value are listed in the bottom of Table 2). Subsequently, the final multiple regression model, with an Enter procedure, including the significantly affecting variables from the previous model (together with lacking dummy variables so that all categories within a certain variable were included), was run. The results from the final multiple regression analysis is presented in Figure 2 and Table 2.Also, group pairs in which slow-release pellets, injections and silastic capsules had been used, respectively, were separately analyzed for association between dosage and EC-ratio. Even if three further large multiple models, as the main model described above, would theoretically have been preferable to control for confounders, the number of groups pairs pertaining to any of the three administration mode categories was too low (in the range of 30–50) to sustain an adequate statistical power. Instead, the relation between dosage and EC-ratio was analyzed with simple linear regression models. The results from these are presented in Figure 3. Three group pairs received silastic capsules containing crystallized 17β-estradiol, and were excluded from this analysis due to difficulty in translating to dissolved concentrations. In two of these three group pairs, estrogens were protective, while no difference was seen in the third group pair.

All statistical calculations were performed in SPSS (Version 20, IBM Corporation, Armonk, NY, USA). P-values <0.05 were considered statistically significant.

### Protocol violations

As mentioned earlier, the variables *Disease* and *Estrogen type* was omitted from the analysis, since too few group pairs included diseased animals or were administered other estrogens than 17β-estradiol, respectively.

One study [40] lacked information about the weight-variable *Number of rats in estrogen treated and control groups*, and the group pair was arbitrarily given the average weight of all other group pairs.

## Competing interests

The authors state that they have no competing interests to report.

## Authors’ contributions

JOS came up with the idea, contributed to the design, performed part of the article inclusion process, performed all analyses and drafted the manuscript. EI contributed to the design, performed part of the article inclusion process and revised the manuscript. Both authors read and approved the final manuscript.
